# Hsa_circRNA_103124 Upregulation in Crohn’s Disease Promotes Cell Proliferation and Inhibits Autophagy by Regulating the Hsa-miR-650/AKT2 Signaling Pathway

**DOI:** 10.3389/fgene.2021.753161

**Published:** 2021-11-05

**Authors:** Juan Yin, Fuyi Tong, Yulan Ye, Tong Hu, Lijuan Xu, Liping Zhang, Jianyun Zhu, Zhi Pang

**Affiliations:** ^1^ Department of Digestive Disease and Nutrition Research Center, The Affiliated Suzhou Hospital of Nanjing Medical University, Suzhou Municipal Hospital, Gusu School, Nanjing Medical University, Suzhou, China; ^2^ The Fifth People’s Hospital of Suzhou, The Affiliated Infectious Diseases Hospital of Soochow University, Suzhou, China; ^3^ Department of Gastroenterology, The Affiliated Suzhou Hospital of Nanjing Medical University, Suzhou Municipal Hospital, Gusu School, Nanjing Medical University, Suzhou, China

**Keywords:** hsa_circRNA_103124, hsa-miR-650, autophagy, Cohn’s disease, AKT2

## Abstract

Circular RNAs (circRNAs) play important roles in the pathogenesis of Crohn’s disease (CD). We discovered that hsa_circRNA_103124 was upregulated in CD patients in our previous study. Nonetheless, the function of hsa_circRNA_103124 is unclear. In this study, hsa_circRNA_103124 was predicted to interact with hsa-miR-650. Gene Ontology (GO) and pathway analyses identified AKT serine/threonine kinase 2 (AKT2) as the downstream target protein of hsa-miR-650. Activated AKT2 inhibits autophagy, but promotes cell proliferation. Recent studies suggest that the inhibition of autophagy is one of the mechanisms of CD pathogenesis. Therefore, we inferred that hsa_circRNA_103124 might regulate autophagy and proliferation by targeting AKT2 as a sponge for hsa-miR-650. Here, quantitative reverse transcription PCR (RT-QPCR) results revealed that upregulated hsa_circRNA_103124 expression in patients with CD was negatively correlated with hsa-miR-650 expression but positively correlated with the white blood cell count and calprotectin levels. TSC complex subunit 1 (TSC1), one of the proteins upstream of autophagy was downregulated in patients with CD. Consisting with the bioinformatics prediction, it was verified that hsa_circRNA_103124 targeted to hsa-miR650 by fluorescence *in situ* hybridization (FISH) and luciferase reporter assays. A hsa-miR-650 inhibitor reversed the promotion of rapamycin-induced autophagy and the inhibition of cell proliferation by the hsa_circRNA_103124 siRNA. However, hsa-miR-650 mimics reversed the inhibition of rapamycin-induced autophagy and the promotion of cell proliferation through hsa_circRNA_103124 overexpression. These results indicate that hsa_circRNA_103124 upregulation in patients with CD promotes cell proliferation and inhibits autophagy by regulating the hsa-miR-650/AKT2 signaling pathway.

## Introduction

The pathogenesis of inflammatory bowel disease (IBD), which includes Crohn’s disease (CD) and ulcerative colitis (UC), is not clearly understood to date. Researchers have universally acknowledged that the complex etiology of IBD is related to the host genetic background, microbial and environmental factors (Ananthakrishnan et al., 2018; Glassner et al., 2020; Piovani et al., 2020; Pittayanon et al., 2020). The sensitivity and specificity of serum biomarkers are limited in diagnosis and treatment of IBD (Zhou et al., 2016). In general, fecal calprotectin (CALP) correlated more closely with the simple endoscopic score for Crohn’s disease (SES-CD) than C-reactive protein (CRP) levels, white blood cell (WBC) counts, and Crohn’s Disease Activity Index (CDAI) (Schoepfer et al., 2010). Fecal CALP is a useful biomarker in the identification and management of patients with CD. However, it performs unsatisfactorily in the diagnosis and treatment of small bowel CD (Vernia et al., 2020). The diagnosis of IBD still relies on invasive endoscopy, while CALP levels, WBC counts and CRP levels only serve as reference biomarkers.

Specific circular RNAs (circRNAs) were discovered to be biomarkers of various diseases (Ouyang et al., 2017; Zhu et al., 2017; Chen et al., 2018; Du et al., 2018; Liu et al., 2018). The function of competing endogenous RNAs (ceRNA) is one of the most important mechanisms that circRNAs may participate in the progression of diseases (Hansen et al., 2013; Suzuki and Tsukahara, 2014; Chen, 2016; Yuan et al., 2018b; Wang et al., 2018). CircRNAs were discovered to be valuable diagnostic biomarkers for CD. Qiao et al. (2019) reported 163 upregulated circRNAs and 55 downregulated circRNAs among the circRNAs in CD tissues by chip screening. We have identified differentially expressed circRNAs (004662, 092520, 102610, and 103124) that are upregulated in the peripheral blood mononuclear cells (PBMCs) of patients with CD using Arraystar Human circRNA Arrays (Yin et al., 2019). Moreover, we conducted an in-depth study on the value of hsa_circRNA_103516 in the clinical diagnosis of CD (Ye et al., 2019). And the mechanism that hsa_circRNA_102610 promotes the epithelial-mesenchymal transition in patients with CD was further studied (Yin et al., 2020). Nonetheless, the function of hsa_circRNA_103124 is unclear.

In this study, the correlation between hsa_circRNA_103124 and CALP levels or WBC counts was assessed in patients with CD. We also predicted by bioinformatics that one of the downstream targets of upregulated hsa_circRNA_103124 in patients with CD was hsa-miR-650. Hsa-miR-650 play different roles in various kinds of diseases. In some reports, hsa-miR-650 targets AKT serine/threonine kinase 2 (AKT2) and inhibits the proliferation, migration and invasion of synovial fibroblasts in individuals with rheumatoid arthritis (Xu et al., 2017). In addition, hsa-miR-650 promotes the inflammation-induced apoptosis of intestinal epithelioid cells (IECs) by targeting NLRP6 (Xu et al., 2019). In colorectal cancer (CRC), the overexpression of hsa-miR-650 promotes the proliferation and migration of CRC cells by targeting inhibitor of growth 4 (ING4) (You et al., 2018). However, no relevant studies on hsa-miR-650 in patients with CD have been reported. In our study, bioinformatics predicted that one of the downstream target genes of hsa-miR-650 is AKT2. This finding is consistent with research results reported by Xu (Xu et al., 2017). The specific signaling pathways downstream of AKT2 are well studied. Activated AKT2 inhibits autophagy, but promotes cell proliferation. Cyclin dependent kinase 2 (CDK2), one of the G1phase regulators, is regulated by AKT2 (Wang et al., 2014). Thus, we inferred that hsa_circRNA_103124 promoted proliferation by regulating the hsa-miR-650/AKT2/CDK2 pathway.

Recently published studies have provided a better understanding of the mechanism of autophagy in IBD. Genome-wide association studies (GWAS) have revealed genes associated with autophagy, such as autophagy-related gene 16 like 1 (*ATG16L1*) and GTPase family M (*IRGM*) (Rioux et al., 2007). Moreover, researchers have postulated that impaired autophagy plays an important role in the pathogenesis of IBD. Dysfunctional autophagy leads to disrupted intestinal epithelial function, gut dysbiosis, defects in antimicrobial peptide secretion by Paneth cells, the endoplasmic reticulum stress response and aberrant immune responses to pathogenic bacteria (Ke et al., 2016; Larabi et al., 2020). TSC complex subunit 1 (TSC1), an upstream protein of mTORC1 and a downstream protein of AKT, plays a critical role in autophagy (Xie et al., 2020). TSC1 deficiency suppresses autophagy (Choi et al., 2018). Therefore, hsa_circRNA_103124 may participate in the inhibition of autophagy by downregulating TSC1 through hsa-miR-650/AKT2. And the expression level of Light Chain 3B (LC3B), which is one of universal-markers of autophagy (Satyavarapu et al., 2018), may change along with variation of hsa_circRNA_103124 in cells.

Thus, according to our previous study, we inferred that upregulated hsa_circRNA_103124 in patients with CD may activate AKT2 by sponging hsa-miR-650. Hsa_circRNA_103124 overexpression may inhibit autophagy and promote cell proliferation. The specific mechanism of hsa_circRNA_103124 in CD pathogenesis requires intensive study.

## Materials and Methods

### Specimen Collection

Patients with CD and healthy donors (HDs) were recruited from 2018 to 2019 at Suzhou Affiliated Hospital of Nanjing Medical University (Suzhou, Jiangsu Province, China). Ethical approval was obtained from the Ethics Committee of Nanjing Medical University. Informed consent was obtained from all participants. Specimens were collected using the method described in our previous study (Yin et al., 2019). Information on all participants is listed in Table 1.

**TABLE 1:** Information on participants.

	CD (*n* = 60)	HC(*n* = 40)
Age, y	37.2 (18–67)	36.6 (23–63)
Sex (M/F) n	42/18	30/10
WBC (10^9^/L)	6.66 (2.9–13.28)	5.99 (3.59–8.47)
CALP (μg/g)	145.8 (7–691)	—
CDAI (Scores)	111.6 (23.72–360.3)	—

WBC, white blood cell Count; CALP, fecal calprotectin; CDAI, Crohn’s disease activity index; y, years; M, male; F, female.

### Analysis of the Relative Expression of hsa_circRNA_103124 and hsa-miR-650 Using RT-QPCR

Total RNA was isolated from PBMCs using the TRIzol Regent (Invitrogen, United States). Complementary DNA (cDNA) was synthesized with PrimeScript RT Master Mix (TaKaRa, Shiga, Japan). The relative expression of hsa_circRNA_103124 in PBMCs was detected using RT-QPCR, with *β*-actin as an internal reference, according to the methods described in our previous studies (Yin et al., 2019; Yin et al., 2020). The relative expression of hsa-miR-650 in PBMCs was detected using RT-QPCR with U6 as an internal reference. In brief, cDNA was synthesized from total RNA with the aid of MMLV Reverse Transcriptase in Hairpin-it™ miRNAs RT-PCR Quantitation Kit (TaKaRa, Shiga, Japan). And RT-QPCR was performed using 2xReal-time PCR Master Mix (SYBR) in Hairpin-it™ miRNAs RT-PCR Quantitation Kit. 2^−△△Ct^ method was used to analyze the relative expression of hsa_circRNA_103124 or hsa-miR-650. The primer sequences are listed in Table 2.

**TABLE 2:** Sequences of primers, siRNAs, microRNA inhibitor, microRNA mimics, and specific probes.

Items	Sequences (5′-3′)
hsa-miR-650 Forward	TGTTCAGGAGGCAGCGCT
hsa-miR-650 Reverse	TAT​GGT​TGT​TCA​CGA​CTC​CTT​CAC
U6 Forward	CGC​TTC​GGC​AGC​ACA​TAT​AC
U6 Reverse	TTC​ACG​AAT​TTG​CGT​GTC​ATC
hsa_circRNA_103124 Forward	TGG​CCC​TTC​TCT​GGA​ATG​TT
hsa_circRNA_103124 Reverse	TGG​AGA​AAT​GTT​TTC​CCT​CTT​GG
β-actin Forward	GTG​GCC​GAG​GAC​TTT​GAT​TG
β-actin Reverse	CCT​GTA​ACA​ACG​CAT​CTC​ATA​TT
si1- circRNA_103124	CAA​ACA​CAA​CCU​CCA​AGA​GTT CUC​UUG​GAG​GUU​GUG​UUU​GTT
si2- circRNA_103124	CCA​CCA​AAC​ACA​ACC​UCC​ATT UGG​AGG​UUG​UGU​UUG​GUG​GTT
si-NC	UUC​UCC​GAA​CGU​GUC​ACG​UTT ACG​UGA​CAC​GUU​CGG​AGA​ATT
miR650 mimics	AGG​AGG​CAG​CGC​CUC​UCA​GGA​C CCU​GAG​AGC​GCU​GCC​UCC​UUU
mimics control	UUC​UCC​GAA​CGU​GUC​ACG​UTT ACG​UGA​CAC​GUU​CGG​AGA​ATT
miR650 Inhibitor	GUC​CUG​AGA​GCG​CUG​CCU​CCU
Inhibitor control	CAG​UAC​UUU​UGU​GUA​GUA​CAA
hsa_circRNA_103124 probe	FAM-CCCTCTTGGAGGTTGTGTTTGGTGGTTTTAAAGTAAACG
hsa-miR-650 probe	CY3-GTCCTGAGAGCGCTGCCTCCT

### Detection of the White Blood Cell Count and Calprotectin Levels

Peripheral blood (1.5 ml) was collected from each patient with CD or healthy control, using a vacuum blood collection tube with EDTA-K2. The WBC count in peripheral blood was detected using an automated hematology analyzer (Sysmex XN-1000, Kobe, Japan). Fresh stool samples (500 mg) were collected from patients with CD. Fecal CALP levels were measured using enzyme-linked immuno sorbent assay (ELISA) (WIZ, BIOTECH, Xiamen, China). Please refer to the manual for specific operational procedures.

### Prediction of the Biological Function of hsa_circRNA_103124

The interaction between circRNA and microRNA was predicted with the Arraystar homemade miRNA target prediction software based on the TargetScan and miRanda databases. The 5 most likely miRNAs to which hsa_circRNA_103124 binds were predicted based on the arrangement of free energy and number of seed sequences. The intersection of the downstream genes of the target miRNAs in the Targetscan, miRDB and miRWalk databases was obtained using Venny 2.1. Gene Ontology (GO) and KEGG pathway analyses were performed to predict the biological function of hsa_circRNA_103124 in DAVID 6.7. Subsequent pairing of target miRNAs and genes was predicted using TargetScan.

### Changes of Expression Level of hsa_circRNA_103124 or hsa-miR-650

Caco2 cells were cultured in Dulbecco’s modified Eagle’s medium (DMEM), and human intestinal epithelial cells (HIECs) were cultured in Roswell Park Memorial Institute 1,640 medium (RPMI 1640 medium), at 37°C with 5% CO2. Over-expression of hsa_circRNA_103124 was induced by transiently transfecting overexpression plasmid pLC5-ciR-circRNA_103124 (Geneseed, Guangzhou, China) in Caco2 cells and HIECs. The downregulation of hsa_circRNA_103124 was induced by transfecting a siRNA (Table 2). The overexpression or downregulation of hsa-miR-650 was induced by RNA mimics or inhibitor (Table 2). Lipofectamine™3000 Reagent (Invitrogen, CA, United States) was used to for the transient transfection of plasmids, siRNA, RNA mimics or inhibitor. In addition, detailed procedures are described in the manual. Relative expression level of hsa_circ_103124 or hsa-miR-650 in Caco2 or HIECs with different treatments in this study were detected by RT-QPCR.

### Cell Proliferation Detected Using Flow Cytometry, Cell Counting Kit-8 Assay and 5-Ethynyl-2′-deoxyuridine Staining

The cell cycle distribution was detected using flow cytometry (FACSCalibur, BD, San Jose, CA, United States) after propidium iodide (PI, Beyotime, Shanghai, China) staining. Cell proliferation was examined using Cell Counting Kit-8 assay (Dojindo, Shanghai, China) and 5-ethynyl-2′-deoxyuridine staining (RiboBio, Guangzhou, Guangdong, China). The detailed methods are described in our previous study (Yin et al., 2020).

### Fluorescence *in situ* Hybridization and Luciferase Reporter Assay

Fluorescence *in situ* hybridization (FISH) was performed to detect the location and expression levels of hsa_circRNA_103124 and hsa-miR-650 using FAM- and CY3-labeled specific probes, respectively (GenePharma, Shanghai, China), in Caco2 cells or HIECs. Please refer to the manual for specific operational procedures. A confocal laser scanning microscope (Carl Zeiss, Göschwitzer Strasse, Jena, Germany) was used to capture the cellular fluorescence images. The sequences of probes used in this experiment are listed in Table 2.

HEK293T cells were cultured in 24-well plates with DMEM, at a density of 2 × 10^4^ cells/ml. Cells were incubated in a humidified atmosphere at 37°C with 5% CO2. Three hundred 1 bp of the hsa-miR-650 response element in hsa_circRNA_103124 and its adjacent sequences were recombined (GenScript, Nanjing, Jiangsu) with psiCHECK-2 (Promega, Madison, WI, United States). A control plasmid including mutated sequences of the hsa-miR-650 response element was inserted into psiCHECK-2. The plasmid and the hsa-miR-650 mimics were transfected into HEK293T cells using Lipofectamine 3,000 Reagent, according to the manual. The Dual-Luciferase® Reporter Assay System (Promega, Madison, WI, United States) was used to detect the activities of Renilla luciferase and firefly luciferase with Biotek Synergy H1(Biotek, Vermont, United States), at 48 h following transfection.

### Counting Acridine Orange -Stained Acidic Autophagic Vesicles

HIECs or Caco2 cells were grown on coverslips in 24-well plates with RPMI 1640 or DMEMmedium at a density of 2 × 10^4^ cells/ml for 24 h. Rapamycin (20 ng/ml) was supplemented to induce autophagy for 24 h. The cells were stained with acridine orange (AO, 2 μg/ml) for 30 min. Fluorescent images of the AO positive cells (orange) were acquired using a confocal laser scanning microscope. The proportion of positively stained cells that underwent autophagy was calculated by normalization to the cells stained green.

### Western-Blotting

The western-blotting methods were described in our previously published paper (Yin et al., 2020). Primary antibodies against the following proteins were used in this study: AKT2 (CST-3063S, Cell Signaling Technology, Danvers, MA, United States), CDK2(CST-2546S, Cell Signaling Technology, Danvers, MA, United States), TSC1(CST-6935S, Cell Signaling Technology, Danvers, MA, United States), microtubule associated protein 1 light chain 3 beta (LC3B, CST-2775S, Cell Signaling Technology, Danvers, MA, United States), and *β*-actin (CST-8457S, Cell Signaling Technology, Danvers, MA, United States).

### Statistical Analysis

GraphPad Prism 5 (GraphPad, La Jolla, CA, United States) was used for data analysis in this study. *p* < 0.05 was considered as a standard to determine statistical significance. Differences between groups were determined using Student’s t-test (between groups) or one-way analysis of variance (ANOVA) (among groups). Pearson’s correlation coefficients were used to describe correlations between variables.

## Results

### Hsa_circRNA_103124 Upregulation in Patients With CD Positively Correlated With the White Blood Cell Count and Calprotectin Levels

**FIGURE 1:**
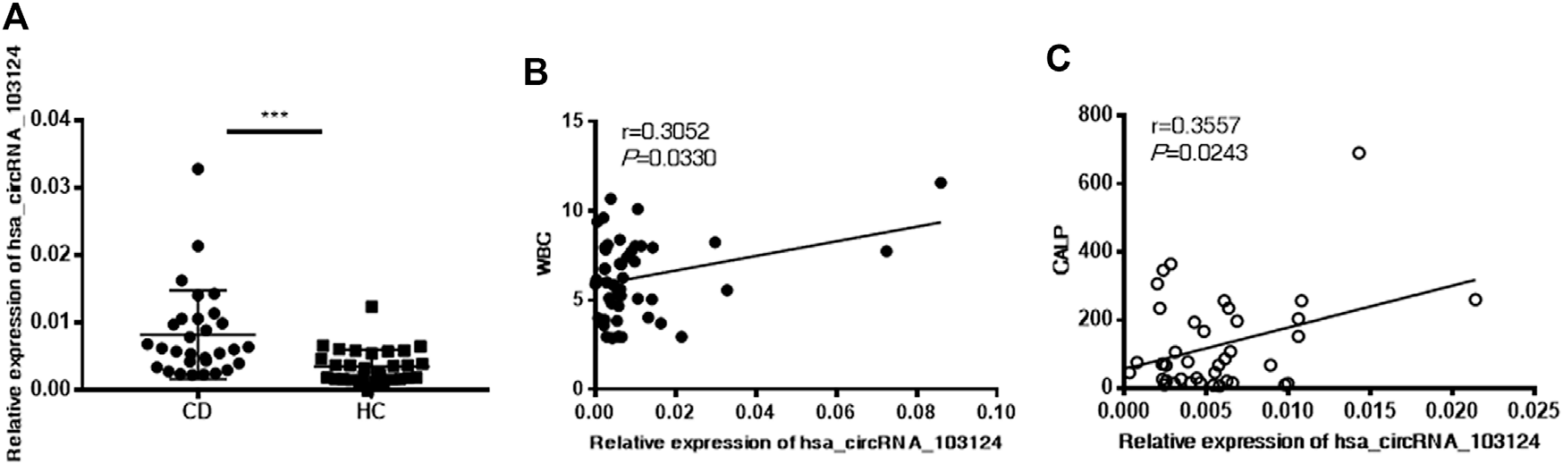
Hsa_circRNA_103124 upregulation in patients with CD positively correlated with the WBC count and CALP level. **(A)** The relative expression level of hsa_circRNA_103124 in peripheral blood mononuclear cells from healthy controls (HC, *n* = 40) or patients with CD (*n* = 60) was detected using RT-QPCR. **(B,C)** The correlation of hsa_circRNA_103124 expression with the WBC count (10^9^/L) or CALP level (μg/g) was analyzed by calculating Pearson’s correlation coefficients. ***p < 0.001.

Hsa_circRNA_103124 was discovered as a potential diagnostic biomarker of CD in our previous study (Yin et al., 2019). The specific correlation between hsa_circRNA_103124 and clinical diagnostic markers of CD was further studied. Hsa_circRNA_103124 was upregulated in patients with CD, which was validated in PBMCs by RT-QPCR (Figure 1A). A correlation analysis was performed between hsa_circRNA_103124 and the WBC count (Figure 1B) or CALP level (Figure 1C) to investigate the possible mechanism underlying the upregulation of hsa_circRNA_103124 in patients with CD. The results showed positive correlations between the expression of hsa_circRNA_103124 in peripheral blood mononuclear cells and WBC counts (*r* = 0.3052, *p* = 0.0330) or CALP (*r* = 0.3557, *p* = 0.0243). The WBC count and CALP level are relatively preferred noninvasive biomarkers for CD diagnosis and treatment as we mentioned in the introduction. Based on this result, hsa_circRNA_103124 potentially served as a diagnostic biomarker for CD.

### Hsa_circRNA_103124 Targeted hsa-miR-650

**FIGURE 2:**
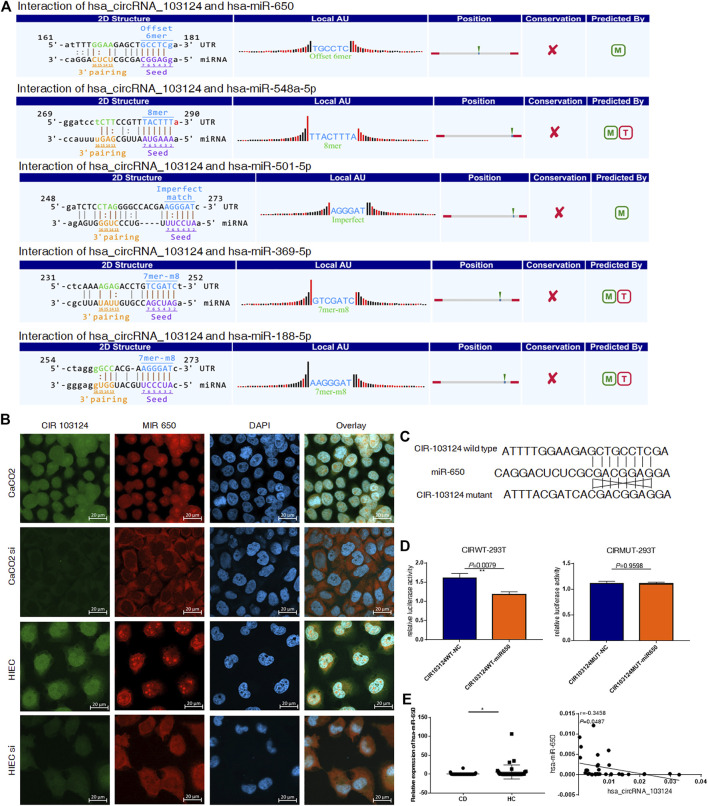
Hsa_circRNA_103124 targeted hsa-miR-650. **(A)** The mircoRNA response element analysis of hsa_circRNA_103124 was conducted using TargetScan and miRanda. **(B)** FISH was performed to detect the location and expression level of hsa_circRNA_103124 and hsa-miR-650 in Caco2 cells and HIECs using FAM- and CY3-labeled specific probes, respectivelyin HEK293T cells. A confocal laser scanning microscope was used to capture the cellular fluorescence images. Bar, 20 μm. **(C)** The sequences of wildtype and corresponding mutated hsa-miR-650 MREs of hsa_circRNA_103124. **(D)** Relative luciferase activity of HEK293T cells cotransfected with the hsa-miR-650 mimics and plasmid including hsa-miR-650 MRE of hsa_circRNA_103124, or HEK 293T cells cotransfected with the hsa-miR-650 mimics and plasmid including mutated sequences of hsa-miR-650 MRE of hsa_circRNA_103124. **(E)** Relative expression of hsa-miR-650 in patients with CD (*n* = 43) or HCs(*n* = 37) was detected using RT-QPCR. The correlation between hsa_circRNA_103124 and hsa-miR-650 expression (*n* = 33) was analyzed by calculating Pearson’s correlation coefficients. *p < 0.05.

According to the MRE analysis, the 5 miRNAs (hsa-miR-650, hsa-miR-548a-5p, hsa-miR-501-5p, hsa-miR-369-5p and hsa-miR-188-5p) that most likely bind to hsa_circRNA_103124 are listed in Figure 2A. In addition, according to the arrangement of free energy and number of seed sequences, hsa-miR-650 is the most likely miRNA. Therefore, FISH was performed to reveal the location and expression of hsa_circRNA_103124 and hsa-miR-650 in HIECs and Caco2 cells. The results **(** Figure 2B **)** showed that hsa_circRNA_103124 and hsa-miR-650 localized in both the cytoplasm and nucleus. When we reduced the expression of hsa_circRNA_103124 transfecting a siRNA in cells, the localization of hsa-miR-650 in the cytoplasm was enhanced. Moreover, luciferase reporter assays (Figures 2C,D) indicated the specificity of the hsa-miR-650 mimics for binding to the hsa_miR_650 response elements in hsa_circRNA_103124. HEK293T cells cotransfected with psiCHECK-2 containing a wild-type hsa-miR-650 MRE of hsa_circRNA_103124 and hsa-miR-650 mimics showed lower relative luminescence intensity. However, cells cotransfected with psiCHECK-2, containing a mutated hsa-miR-650 MRE of hsa_circRNA_103124 and hsa-miR-650 mimics showed no significant change in the relative luminescence intensity. Therefore, we inferred that hsa-miR-650 bound to the MRE of hsa_circRNA_103124. In patients with CD, the relative expression of hsa-miR-650 was downregulated (*p* = 0.043) compared with that in healthy controls. Moreover, hsa_circRNA_103124 expression negatively correlated with hsa-miR-650 expression (Figure 2E). Therefore, we inferred that hsa_circRNA_103124 might play a negative regulatory role in hsa-miR-650 expression. Correspondingly, the function of downstream proteins of hsa-miR-650 could be regulated.

### Hsa_circRNA_103124 Targeted AKT2 by Sponging hsa-miR-650

**FIGURE 3:**
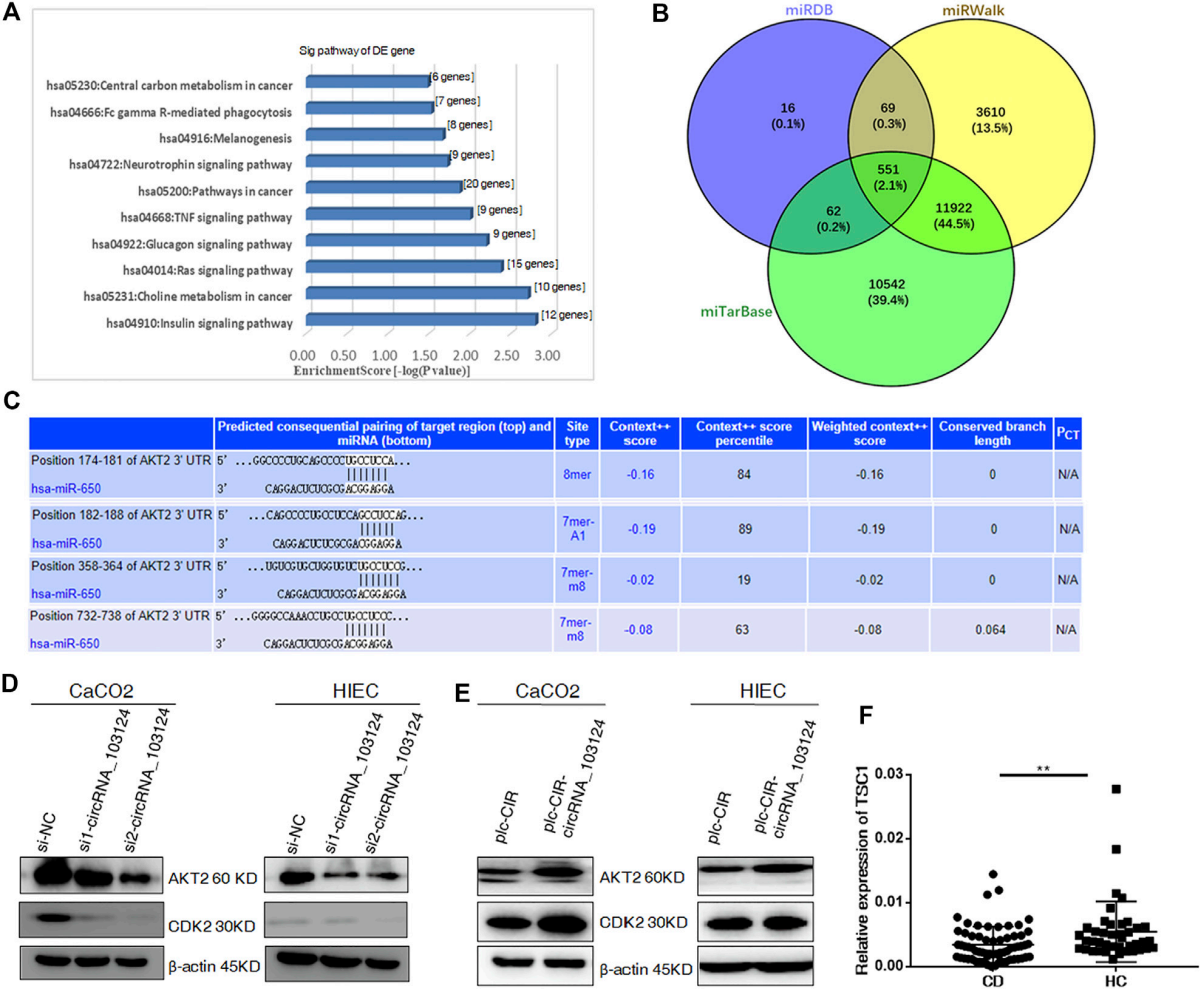
Hsa_circRNA_103124 targets AKT2 by sponging hsa-miR-650. **(A)** GO and KEGG pathway analyses were performed to predict the biological functions of hsa_circRNA_103124 using DAVID 6.7. **(B)** The intersection of the downstream genes of hsa-miR-650 in the miTarBase, miRDB and miRWalk databases was obtain in Venny 2.1. **(C)** Subsequent pairing of target miRNAs and genes was predicted using TargetScan. **(D)** Relative expression of AKT2 and CDK2 was detected using western-blotting following the transfection of the hsa_circRNA_103124 siRNA, and normalized to *β*-actin. si-NC, cells transfected with the negative control of hsa_circRNA_103124 siRNA; si1-circRNA_103124, si2-circRNA_103124, cells transfected with the siRNA targeting hsa_circRNA_103124. **(E)** Relative expression of AKT2 and CDK2 was detected using western-blotting following hsa_circRNA_103124 overexpression, and normalized to *β*-actin. plc-CIR, cells transfected with the plasmid control plc-CIR; plc-CIR-circRNA_103124, cells transfected with the hsa_circRNA_103124 overexpression plasmid plc-CIR-circRNA_103124. **(F)** Relative expression of TSC1 in patients with CD (*n* = 60) and HCs(*n* = 40) was performed by RT-QPCR. **p < 0.01.

GO and KEGG pathway analyses indicated that hsa-miR-650 participates in pathways in cancer and TNF signaling (Figure 3A). The Venn diagram showed 551 common downstream genes of hsa-miR-650 identified in Targetscan, miRDB and miRWalk databases (Figure 3B), which contributed to the abovementioned pathways in which hsa-miR-650 played roles. Four binding sites for hsa-miR-650 in 3′UTR of AKT2 were predicted by TargetScan (Figure 3C). The protein expression levels of AKT2 and CDK2 were down-regulated in HIECs and Caco2 in response to interference with hsa_circRNA_103124 expression by siRNAs (Figure 3D). AKT2 and CDK2 were upregulated following hsa_circRNA_103124 overexpression (Figure 3E). TSC1 (a downstream protein of AKT2) was expressed at lower levels in patients with CD (Figure 3F). Considering the aforementioned results that hsa_circRNA_103124 targeted hsa-miR-650, we inferred that hsa_circRNA_103124 regulated AKT2 and its downstream proteins CDK2 and TSC1 by sponging hsa-miR-650. However, further studies should be performed on the specific roles that hsa-miR-650 may play in the functions of hsa_circRNA_103124.

### The hsa-miR-650 Inhibitor Reversed the Inhibitory Effect of hsa_circRNA_103124 siRNA on Cell Proliferation

One of the most important biological functions of AKT2 is regulation of proliferation. Therefore, 5-Ethynyl-2′-deoxyuridine (EdU) staining, Cell Counting Kit-8 (CCK-8) assay and the cell cycle distribution test were performed to study the status of cell proliferation following changes in hsa_circ_103124 expression. In EdU staining, thymine in the DNA of proliferating cells was replaced with EdU, which was stained by Apollo, as shown in red. The ratio of red to blue cells, represents the percentage of proliferating cells. In the CCK-8 assay, the OD450 absorbance value showed cell viability. The cell cycle distribution was analyzed using flow cytometry revealed the proportion of cells in G1, S or G2 phases.

**FIGURE 4:**
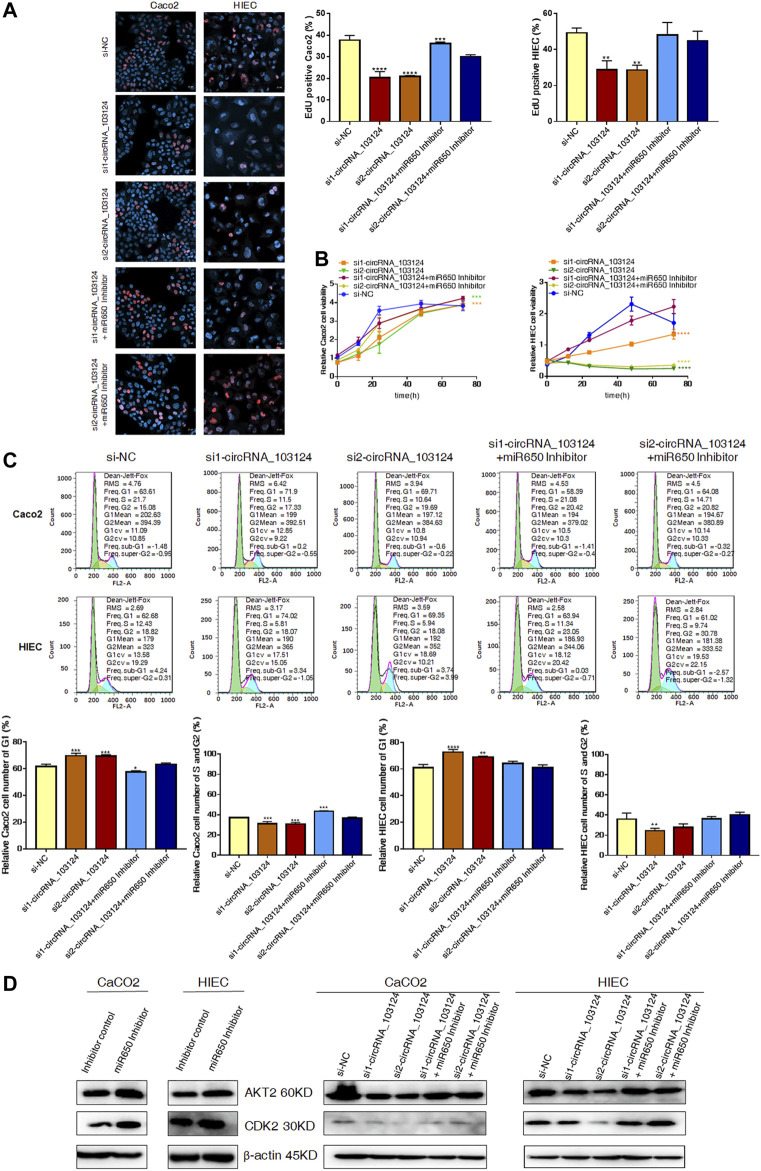
The hsa-miR-650 inhibitor rescued the inhibition of cell proliferation induced by the hsa_circRNA_103124 siRNA. **(A,B)** Cell proliferation was assessed using 5-ethynyl-2′-deoxyuridine staining and the CCK-8 assay. **(C)** The cell cycle distribution was detected using flow cytometry following propidium iodide staining. **(D)** Relative expression of AKT2 and CDK2 was detected using western-blotting, and normalized to *β*-actin. si-NC, cells transfected with negative control of hsa_circRNA_103124 siRNA. si1-circRNA_103124, si2-circRNA_103124, cells transfected with the hsa_circRNA_103124 siRNA; Inhibitor control, cells transfected with the negative control of the hsa-miR-650 inhibitor. si1-circRNA_103124 + miR650 Inhibitor, si2-circRNA_103124 + miR650 Inhibitor, cells cotransfected with hsa_circRNA_103124 siRNA and hsa-miR-650 inhibitor. Inhibitor control, cells transfected with the negative control of hsa-miR-650 inhibitor. miR650 Inhibitor, cells transfected with hsa-miR-650 inhibitor.

The expression of hsa_circRNA_103124 was downregulated in Caco2 cells or HIECs transfected with hsa_circRNA_103124 siRNAs (Supplementary Figure S1A). The results of EdU staining (Figure 4A), the CCK-8 assay (Figure 4B), and cell cycle distribution detection using flow cytometry (Figure 4C) indicated that hsa_circRNA_103124 downregulation in Caco2 cells and HIECs inhibited cell proliferation. A lower ratio of red to blue cells was observed by EdU staining (Figure 4A). A smaller OD450 absorbance value was detected at each time point (Figure 4B). Notably, siRNA mediated depletion of hsa_circRNA_103124 led to an increase in the numbers of Caco2 and HIEC cells in G1 phase and a concomitant decrease in the numbers of cells in S and G2 phases (Figure 4C). Furthermore, western-blotting showed that the inhibition of hsa_circRNA_103124 was accompanied by downregulation of AKT2 and CDK2 (Figure 4D). Meanwhile, a rescue experiment was carried out by cotransfecting of a hsa-miR-650 inhibitor with the hsa_circRNA_103124 siRNA. Hsa-miR-650 inhibitor couldn’t rescue the expression of hsa_circRNA_103124 (Supplementary Figure S1A). And there was no stable significant change of relative expression of hsa-miR-650 in Caco2 cells or HIECs transfected with hsa_circRNA_103124 siRNAs (Supplementary Figure S1B). However, the results of EdU staining (Figure 4A), the CCK-8 assay (Figure 4B) and cell cycle distribution detection using flow cytometry (Figure 4C) suggested that the hsa-miR-650 inhibitor reversed the effect hsa_circRNA_103124 on restoring cell proliferation. Moreover, the expression of AKT2 and CDK2 was upregulated by the hsa-miR-650 inhibitor (Figure 4D), with decreased expression of hsa-miR-650 in Caco2 cells and HIECs (Supplementary Figure S1C). Nevertheless, there was no significant change of relative expression of hsa_circRNA_103124 in Caco2 cells and HIECs transfected with hsa-miR-650 inhibitor (Supplementary Figure S1D). Therefore, we considered that CDK2, one of the G1 phase regulators, was regulated by AKT2 and its upstream molecules hsa-miR-650/hsa_circRNA_103124. The hsa-miR-650 inhibitor rescued the inhibitory effect of the hsa_circRNA_103124 siRNA on cell proliferation, along with corresponding changes of AKT2 and CDK2 in expression levels.

### Hsa-miR-650 Mimics Reversed the Induction of Cell Proliferation by hsa_circRNA_103124 Overexpression

**FIGURE 5:**
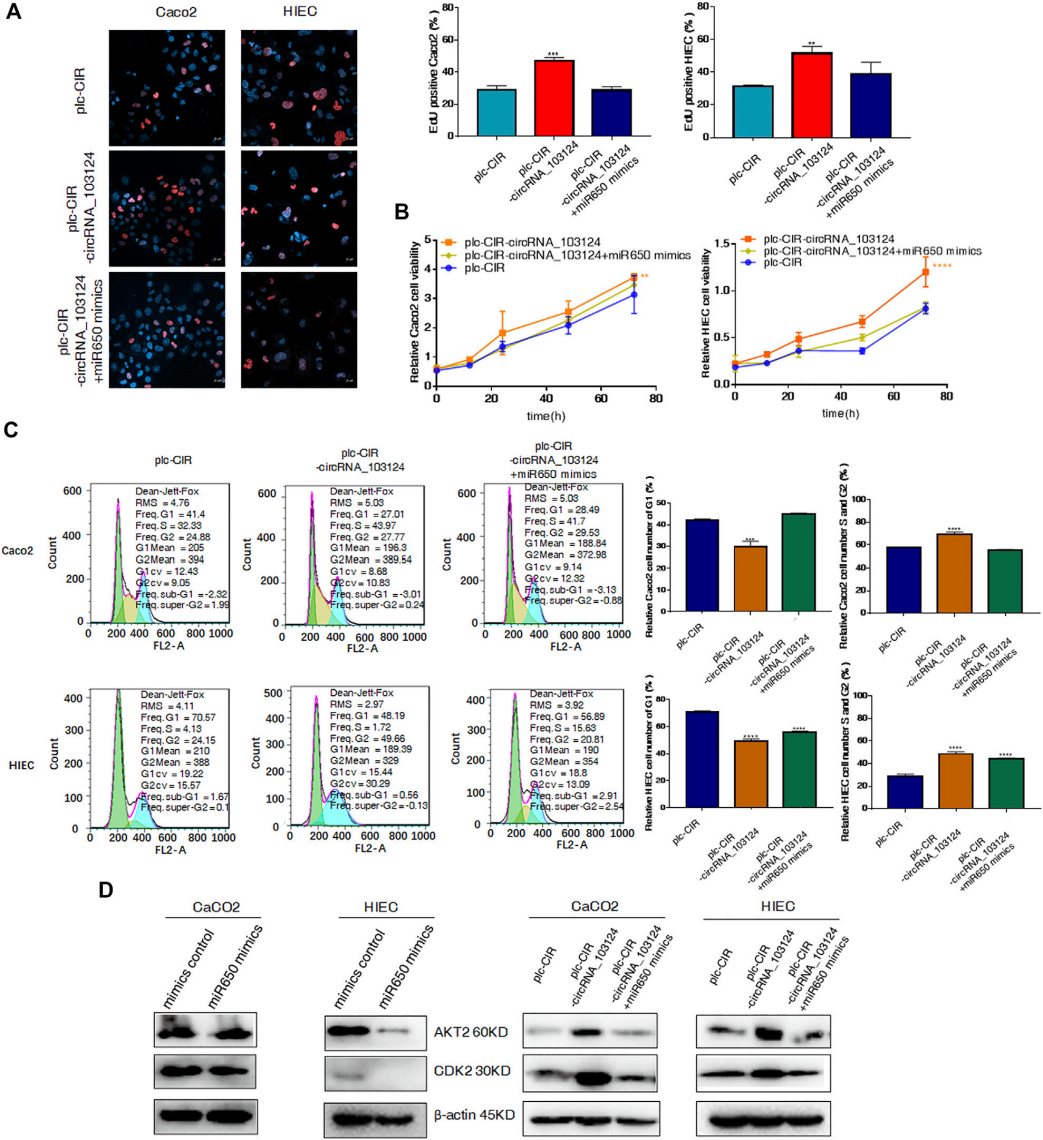
Hsa-miR-650 mimics reversed the promotion of cell proliferation induced by hsa_circRNA_103124 overexpression. **(A–C)** Cell proliferation was tested using 5-ethynyl-2′-deoxyuridine staining, the CCK-8 assay and flow cytometry. **(D)** Relative expression of AKT2 and CDK2 was detected using western-blotting, and normalized to *β*-actin. plc-CIR, cells transfected with plasmid control plc-CIR; plc-CIR-circRNA_103124, cells transfected with the hsa_circRNA_103124 overexpressed plasmid plc-CIR-circRNA_103124; plc-CIR-circRNA_103124 + miR650 mimics, cells cotransfected with the hsa_circRNA_103124 overexpressed plasmid plc-CIR-circRNA_103124 and hsa-miR-650 mimics. mimics control, cells transfected with the negative control of hsa-miR-650 mimics. miR650 mimics, cells transfected with hsa-miR-650 mimics.

We confirmed that hsa_circRNA_103124 overexpression in Caco2 cells and HIECs promoted proliferation by performing EdU staining (Figure 5A), the CCK-8 assay (Figure 5B) and determining the cell cycle distribution (Figure 5C). With hsa_circRNA_103124 overexpressed (Supplementary Figure S1E), a higher ratio of red to blue cells was observed by EdU staining (Figure 5A). A greater OD450 absorbance value was detected at each time point (Figure 5B). The relative cell numbers of Caco2 cells and HIECs in G1 phase were decreased. While the relative cell numbers of Caco2 cells and HIECs in S and G2 phases were increased (Figure 5C). Furthermore, the expression of AKT2 and CDK2 was upregulated following the overexpression of hsa_circRNA_103124 (Figure 5D). Correspondingly, hsa-miR-650 mimics were cotransfected with hsa_circRNA_103124 in Caco2 cells and HIECs in rescue experiments. Hsa-miR-650 mimics couldn’t reverse the upregulation of hsa_circRNA_103124 in cells transfected with plc-CIR-circRNA_103124 (Supplementary Figure S1E). And the expression of hsa-miR-650 wasn’t increased with hsa_circRNA_103124 overexpressed (Supplementary Figure S1F). However, the expression of hsa_circRNA_103124 was down-regulated in Caco2 cells and HIECs transfected with hsa-miR-650 mimics merely (Supplementary Figures S1G,H). The results of EdU staining (Figure 5A), the CCK-8 assay (Figure 5B) and cell cycle distribution detection using flow cytometry (Figure 5C) indicated that the hsa-miR-650 mimics reversed the increase in cell proliferation induced by the overexpression of hsa_circRNA_103124. In addition, the expression of AKT2 and CDK2 was downregulated by hsa-miR-650 mimics. Meanwhile, hsa-miR-650 mimics reversed the upregulation effect of hsa_circRNA_103124 on AKT2 and CDK2 (Figure 5D). Therefore, overexpression of hsa_circRNA_103124 promoted proliferation of Caco2 cells and HIECs. While hsa-miR-650 mimics could rescue this effect. And we inferred that hsa_circRNA_103124 regulated the functions of hsa-miR-650 in a ceRNA manner, rather than by regulating the expression of hsa-miR-650.

### The hsa-miR-650 Inhibitor Reversed the Induction Effect of hsa_circRNA_103124 siRNA on Autophagy

**FIGURE 6:**
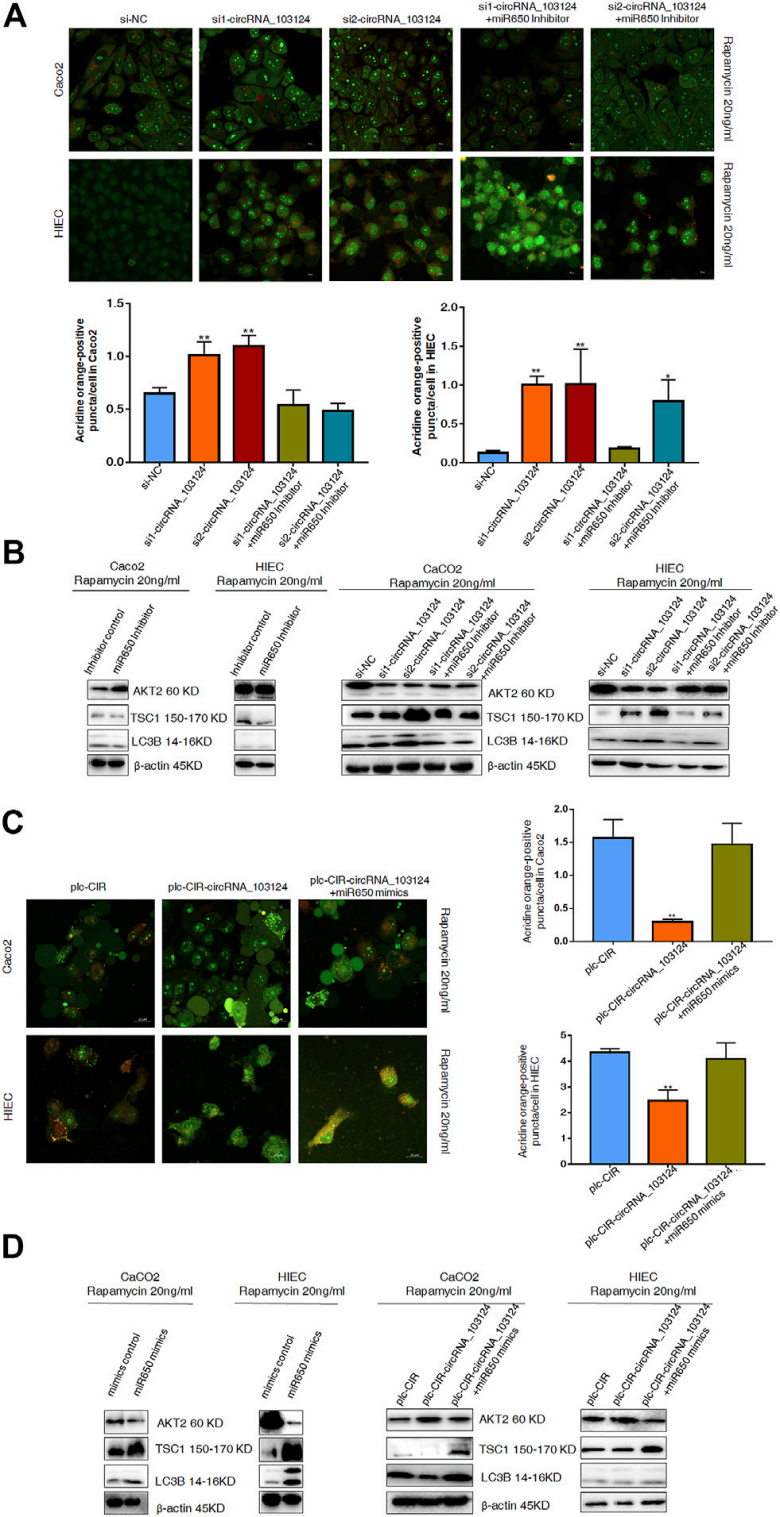
Hsa-miR-650 rescued the inhibitory effects on autophagy induced by hsa_circRNA_103124 overexpression. **(A)** Autophagy was induced by rapamycin (20 ng/ml) in HIECs and Caco2 following transfection of the hsa_circRNA_103124 siRNA. Fluorescent images of acridine orange (AO) positive cells (in orange) were acquired using a confocal laser scanning microscope. The proportion of positively stained cells (orange) that underwent autophagy was calculated relative cells stained green. **(B)** Relative expression of AKT2, TSC1 and LC3B was detected using western-blotting and normalized to *β*-actin. si-NC, cells transfected with negative control of hsa_circRNA_103124 siRNA. si1-circRNA_103124, si2-circRNA_103124, cells transfected with the hsa_circRNA_103124 siRNA; si1-circRNA_103124 + miR650 Inhibitor, si2-circRNA_103124 + miR650 Inhibitor, cells cotransfected with hsa_circRNA_103124 siRNA and hsa-miR-650 inhibitor. Inhibitor control, cells transfected with the negative control of hsa-miR-650 inhibitor. miR650 Inhibitor, cells transfected with hsa-miR-650 inhibitor. **(C)** Autophagy was induced by rapamycin (20 ng/ml) in HIECs and Caco2 cells following hsa_circRNA_103124 overexpression. Fluorescent images of AO-positive cells (orange) were acquired using a confocal laser scanning microscope. The proportion of positively stained cells that underwent autophagy was calculated relative to cells stained green. **(D)** Relative expression of AKT2, TSC1 and LC3B was detected using western-blotting and normalized to *β*-actin. plc-CIR, cells transfected with plasmid control plc-CIR; plc-CIR-circRNA_103124, cells transfected with the hsa_circRNA_103124 overexpressed plasmid plc-CIR-circRNA_103124; plc-CIR-circRNA_103124 + miR650 mimics, cells cotransfected with the hsa_circRNA_103124 overexpressed plasmid plc-CIR-circRNA_103124 and hsa-miR-650 mimics. mimics control, cells transfected with the negative control of hsa-miR-650 mimics. miR650 mimics, cells transfected with hsa-miR-650 mimics.

Impaired autophagy plays an important role in the pathogenesis of IBD (Larabi et al., 2020). AKT2 is one of the most important proteins that regulate autophagy (Xie et al., 2020). TSC1 and LC3B are downstream proteins of AKT2 (Satyavarapu et al., 2018; Xie et al., 2020). Therefore, autophagy was induced by rapamycin (20 ng/ml) in Caco2 cells and HIECs to study the function of hsa_circRNA_103124 on autophagy. In the present study, hsa_circRNA_103124 downregulation by siRNAs promoted rapamycin-induced autophagy, as shown by significantly increased numbers of AO-positive particles (orange), (Figure 6A). Moreover, AKT2 expression was downregulated. The expression of TSC1 and LC3B was upregulated (Figure 6B). However, the hsa-miR-650 inhibitor reversed these effects, leading to opposite effects on protein expression (Figure 6B) and decreased numbers of AO-positive particles (Figure 6A).

### Hsa-miR-650 Mimics Reversed the Inhibition of Rapamycin-Induced Autophagy by hsa_circRNA_103124

The overexpression of hsa_circRNA_103124 in Caco2 cells and HIECs inhibited rapamycin-induced autophagy and reduced the number of AO-positive particles (Figure 6C). The expression of AKT2 expression was upregulated, while the expression of TSC1 and LC3B was downregulated (Figure 6D). However, when hsa-miR-650 mimics were cotransfected, autophagy was restored, as shown by significantly increased numbers of AO-positive particles (orange), (Figure 6C). The expression of AKT2 was downregulated, and the expression of TSC1 and LC3B was upregulated (Figure 6D). Thus, hsa_circRNA_103124 inhibited autophagy in a hsa-miR-650 dependent way.

## Discussion

CircRNAs are considered important regulatory molecules in large numbers of biological processes involved in various diseases (Lin et al., 2020). An increasing number of studies have reported that circRNAs play important roles in the pathogenesis of IBD. Upregulated hsa_circRNA_102685, which was identified in colonic tissues from patients with CD using microarray, is possibly involved in the apoptosis, p53 and Toll-like receptor signaling pathways (Qiao et al., 2019). Genome wide association studies have reported that single nucleotide polymorphisms (SNPs) in circQTL are significantly enriched in patients with IBD. CircRNA formation might be influenced by circQTL SNPs which may alter canonical splicing (Liu et al., 2019). Moreover, it was discovered that circRNAs are involved in the self-renewal ability of intestinal stem cells, regulating expression of autophagy-related genes in intestinal epithelium. (Yuan et al., 2018a; Zhu et al., 2019; Li et al., 2020). Our previous studies have identified several circRNAs (004662, 092520, 102610, and 103124) that are upregulated in PBMCs as latent biomarkers for the diagnosis of CD using microarray (Yin et al., 2019). In addition, further studies showed that upregulated hsa_circRNA_102610 in patients with CD promotes the transforming growth factor-β1 induced epithelial-mesenchymal transition by sponging hsa-miR-130a-3p (Yin et al., 2020). However, the specific roles that hsa_circRNA_103124 may play in the pathogenesis of CD is unclear.

In the present study, the mechanism that hsa_circRNA_103124 may participate in the pathogenesis of CD was explored. The results in this study demonstrated positive correlations of hsa_circRNA_103124 with WBC count and CALP level in patients with CD. Fecal CALP and WBC are useful biomarkers in the diagnosis of patients with CD (Schoepfer et al., 2010). Therefore, hsa_circRNA_103124 could be a potential biomarker for CD diagnosis. And it may regulate inflammatory response of patients with CD. Meanwhile, hsa-miR-650 was predicted to be a target of hsa_circRNA_103124. A negative correlation of hsa_circRNA_103124 with hsa-miR-650 was discovered by RT-QPCR in patients with CD. FISH proved that hsa_circRNA_103124 and hsa-miR-650 were expressed colocalized in HIECs and Caco2 cells. And we found that the MRE of hsa_circRNA_103124 directly bound to hsa-miR-650 mimics by luciferase reporter assays. Thus, the possible ceRNA function of hsa_circRNA_103124 was further confirmed.

GO and KEGG pathway analyses showed that AKT2 is a downstream target of hsa-miR-650. Hsa-miR-650 was reported to suppress proliferation, migration and invasion in individuals with rheumatoid arthritis by targeting AKT2 (Xu et al., 2017). One of the crucial biological functions regulated by AKT2 is autophagy. TSC1 is an important regulator in AKT/mTORC1 pathway (Xie et al., 2020). TSC1 deficiency suppresses autophagy (Choi et al., 2018). Although a significant negative correlation was not observed between the expression of TSC1 and hsa_circRNA_103124, TSC1 was downregulated in patients with CD, as predicted. Crosstalk between the inflammasome and autophagy has been observed (Cosin-Roger et al., 2017). Inflammasome activation is inhibited by autophagy, while autophagy activation is regulated by ROS and inflammasomes. NLRP3 was identified as a novel binding partner of the autophagy inhibitor mTOR (Cosin-Roger et al., 2017). Autophagy plays key roles in intestinal homeostasis, regulation of innate and adaptive immunity, and host defense against intestinal pathogens. The mechanism by which impaired autophagy participates in the pathogenesis of IBD is a current research hotspot. GWAS discovered genes related to autophagy in patients with CD, including ATG16L1 and IRGM (Rioux et al., 2007). The *ATG16L1 T300A* variant results in defective autophagy-induction, which disrupts the homeostasis of the intestinal epithelium, leading to disordered inflammatory immune responses (Larabi et al., 2020). Therefore, the specific functions of hsa_circRNA_103124 on proliferation and autophagy were further studied in HIECs and Caco2 cells.

In our study, overexpression of hsa_circRNA_103124 in Caco2 cells and HIECs promoted cell proliferation. The rapamycin induced autophagy was inhibited by overexpression of hsa_circRNA_103124, as well. The expression of AKT2 or CDK2 was increased in cells with hsa_circRNA_103124 overexpressed, while the expression of TSC1 or LC3B was downregulated. Hsa-miR-650 mimics reversed the effects of hsa_circRNA_103124 overexpression. The results achieved with the hsa_circRNA_103124 siRNA and hsa-miR-650 inhibitor were consistent with these findings. Therefore, we inferred that hsa_circRNA_103124 acted as a ceRNA by targeting hsa-miR-650 to promote cell proliferation and inhibit autophagy. Autophagy inhibition in CD could induce disordered inflammatory immune responses, which may be the probable mechanism that hsa_circRNA_103124 participates in CD progression.

However, several limitations exist in this study. First, our research was conducted only on clinical samples and cells. *In vivo* studies with animal models should be carried out to further prove the role of hsa_circRNA_103124 in the mechanism of CD pathogenesis. Second, this research included only samples from patients with CD. Further research is needed to determine whether hsa_circRNA_103124 participates in the progression of UC, which is another common type of IBD. Third, the mechanism by which hsa_circRNA_103124 participates in clinical CD *via* autophagy requires in-depth investigation. Fourth, the Arraystar chip was applied in this study to discover differentially expressed circRNAs in patients with CD. Nonetheless, many less abundant circRNAs or circRNAs expressed at low levels may be missed due to the limitation of this method. Deep RNA sequencing may be an ideal method to capture maximum circRNAs, which will be conducted in our future research.

## Conclusion

**FIGURE 7:**
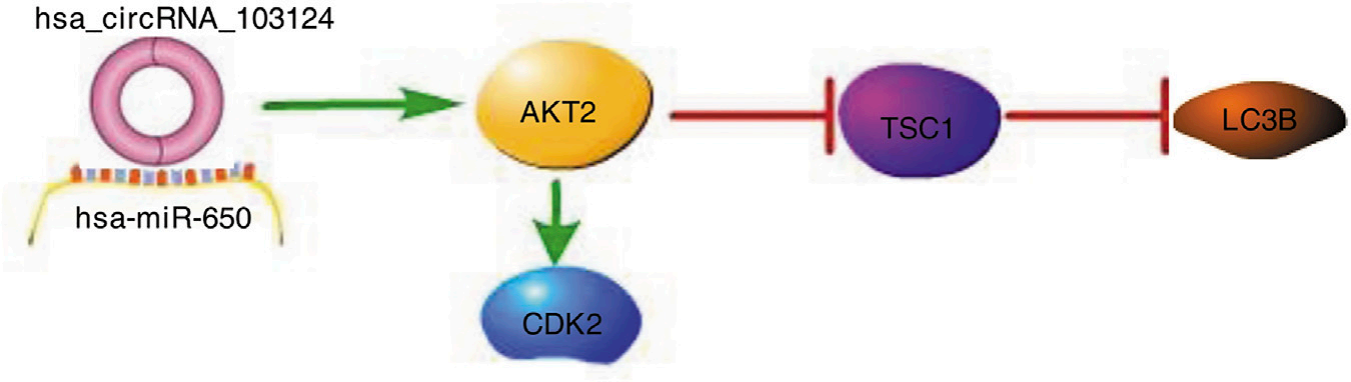
Hsa_circRNA_103124 upregulated in patients with CD inhibits autophagy by targeting AKT2 and sponging hsa-miR-650. A schematic diagram of the proposed mechanism by which that hsa_circRNA_103124 upregulation in patients with CD inhibits autophagy by targeting AKT2 and sponging hsa-miR-650. Pathway Builder Tool 2.0 was used to create this diagram. Upregulated hsa_circRNA_103124 in patients with CD targets to hsa-miR-650. AKT2, a downstream protein of hsa-miR-650, is upregulated, which leads to increased expression of CDK2 and reduced expression of TSC1 and LC3B. Therefore, the proliferation of intestinal epithelial cells is increased, while autophagy is inhibited.

In conclusion, we first studied the specific mechanism by which hsa_circRNA_103124 participates in CD. Based on the research results from this study, hsa_circRNA_103124 inhibited rapamycin induced autophagy by targeting the AKT2/TSC1/LC3B pathway as a sponge of hsa-miR-650. The proliferation of HIECs and Caco2 cells was promoted by hsa_circRNA_103124 in a hsa-miR-650/AKT2/CDK2 dependent manner (Figure 7).

## Data Availability

The data that support the findings of this study are available from the corresponding author, without reservation.
