# Supplementary figures and images for: Hsa_circRNA_103124 Upregulation in Crohn’s Disease Promotes Cell Proliferation and Inhibits Autophagy by Regulating the Hsa-miR-650/AKT2 Signaling Pathway
Source: Front Genet. 2021 Nov 5;12:753161. doi: 10.3389/fgene.2021.753161 (PMC8602894; doi:10.3389/fgene.2021.753161)

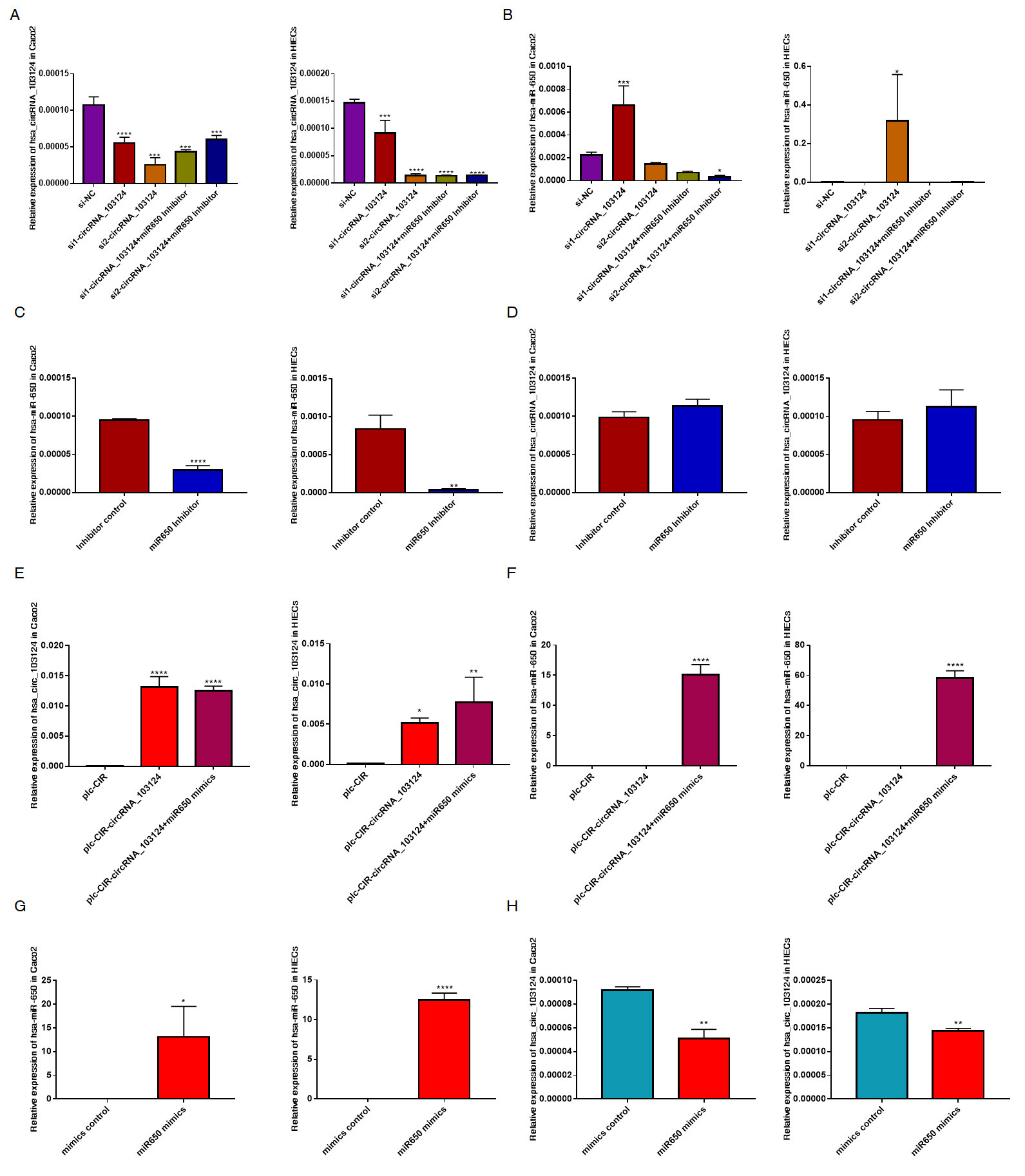

Supplement: Supplementary file 1 [file Image1.TIF]
